# Pediatric Palliative Care: Insights into Assessment Tools and Review Instruments

**DOI:** 10.3390/children10081406

**Published:** 2023-08-17

**Authors:** Simonetta Papa, Anna Mercante, Luca Giacomelli, Franca Benini

**Affiliations:** 1 Polistudium SRL, 20121 Milan, Italy; simonetta.papa@polistudium.it (S.P.); luca.giacomelli@polistudium.it (L.G.); 2 Child and Adolescent Neuropsychiatry Unit, Ospedale San Bortolo, 36100 Vicenza, Italy; 3 Pediatric Palliative Care, Pain Service, Department of Women’s and Children’s Health, University of Padua, 35122 Padua, Italy; franca.benini@aopd.veneto.it

**Keywords:** pediatric palliative care, assessment tools, quality of life, pain, symptoms

## Abstract

The proper assessment of needs and outcomes in pediatric palliative care (PPC) is imperative to ensure the best possible service to patients and families. However, given the multidimensional nature of PPC, the low number of patients in this setting, the heterogeneity of diseases, the presence of cognitive impairment in many patients, and the physiological development of children, outcomes can be complex and difficult to measure. Consequently, in this context, the use of standardized and validated tools to assess the needs of children and families, to assess symptom severity, and to estimate the quality of PPC service represent a current need. Even if efforts have been made to standardize approaches and tools for palliative care in adults, to our knowledge, a similar comprehensive assessment of PPC has not yet been conducted to date. This narrative review provides an overview and discusses the evaluation of tools currently applied in PPC, with an educational intent for healthcare providers. We found that several instruments are available to assess different dimensions of PPC. We proposed a classification into eligibility tools, patient and family needs assessment tools, and care assessment tools. At present, two main eligibility tools exist, the PaPaS Scale and the ACCAPED Scale questionnaire. Most of the tools for patient and family needs assessment have not been specifically validated in the PPC setting, and many may be more readily applied in research settings rather than in daily practice. Similar considerations can be made for tools assessing QoL, while tools assessing PPC service quality seem to be easily applied. Efforts to develop new specific tools and validate existing ones are undoubtedly advocated. However, in the patient’s best interest, PPC healthcare providers should start using available tools, regardless of their validation status.

## 1. Introduction

In the pediatric setting, palliative care (PC) is defined as ‘the active total care of the child’s body, mind and spirit, while also giving support to the family’ [1]. Remarkably, access to pediatric PC (PPC) is a recognized right for eligible children and their families [2].

PPC should be introduced when an incurable or life-threatening illness is diagnosed and should not be restricted to the end-of-life period. Therefore, as we pointed out in our previously published worldwide standards for PPC, assessing child and family needs and monitoring the quality of PPC are fundamental to continuously improving care based on objective information and data [2]. Using standardized and validated tools is fundamental to improving the quality and equity of care and to providing robust evidence in the PPC field [3].

Even if the need for PPC is unquestionable, there is limited evidence of the quality or outcomes of the care provided [3]. To this aim, the use of standardized and validated tools when possible, validated for the specific language and culture, to assess and measure the needs of children and families in the different phases of the disease trajectory, to assess the symptom severity, and to provide an estimate of PPC service quality represents a current need [2].

Efforts have been made to standardize approaches and assessment tools for PC in adults [4]. However, to our knowledge, a similar comprehensive assessment in the field of PPC has not yet been conducted [3]. Indeed, because of the multidimensional nature of PPC, outcomes can be complex and difficult to measure. Furthermore, the relatively low number of patients in the PPC setting, the heterogeneity of diseases, the presence of cognitive impairment in many patients, and the physiological development of children make any evaluation much more challenging.

To support and promote the use of available tools, in this narrative review, we provide an overview and discuss the evaluation of tools currently applied in PPC, with an educational intent for the healthcare providers (HCPs) operating in this setting. A classification of these tools is also proposed to facilitate the immediate understanding of the reader and the application of those tools in clinical practice.

## 2. Methodology

Two clinicians with extensive expertise in PPC (AM, FB), supported by two researchers with proven experience in this field (SP, LG), took part in this project, aimed at providing a narrative review for HCPs with a detailed and comprehensive overview of evaluation tools currently applied in PPC.

To this end, several interrelated PubMed searches were conducted by the authors, with no limitations in terms of publication date and language. Different combinations of pertinent keywords (e.g., PPC and outcome measurement; PPC and evaluation tools; PPC and needs assessment; PPC and quality of care) were used. Documents from the authors’ personal collection of literature were also considered. The papers were selected for inclusion based on their relevance to the topic, as unanimously judged by the authors. Any disagreements during the paper selection process were resolved by internal discussion among the authors. The research was last updated at the end of June 2023.

For the sake of clarity, retrieved tools were classified according to their topics and areas of application. Due to the heterogeneity of the available tools and the educational intent of this paper, we did not conduct a more formal evaluation of the available tools using any standardized method (e.g., by the COSMIN approach [5]).

## 3. Classification of Tools

Based on our literature analysis, the classification of assessment tools in PPC can be proposed (Table 1). The available tools are then commented on and presented according to this classification, mainly for educational purposes.

### 3.1. Eligibility Tools

Eligibility tools allow the early identification of children eligible for PPC through assessment of their needs. They also allow children to be referred to the services that best meet the child’s and family’s needs (palliative approach, generalized or specialized PPC). They are applied at the initiation of the PPC service but can also be applied along the disease trajectory when changes occur that may suggest referral to a different service.

### 3.2. Patient and Family Needs Assessment Tools

Patient and family needs assessment tools assess the severity and management of needs—defined as physical, psychological, social, spiritual, and ethical according to the recently developed international standards for PPC [2]. These tools must be applied throughout the PPC trajectory.

### 3.3. Care Assessment Tools

Care assessment tools assess the quality of services and the effectiveness of the PPC programs and are repeated throughout the PPC trajectory as necessary.

## 4. Eligibility Tools

The prompt institution of PPC is crucial to optimize clinical outcomes and ensure the best possible quality of life (QoL) for the child and family [2]. Currently, the main tools for the early identification of children eligible for PPC and referral to the services that can best meet their needs are the Pediatric Palliative Screening Scale (PaPaS Scale) [6,7] and the Assessment Form for Complex Clinical Needs in Pediatrics (ACCAPED Scale) [8,9]. The PaPaS Scale assesses the care burden on the child and caregivers, while the ACCAPED Scale is a form that allows for quantifying the complexity of care by analyzing clinical needs. For both scales, a score is assigned to each individual item; combining the scores allows the child to be ranked and the best PPC service to be identified. The ‘surprise question’ can also be considered in this group (Table 2).

### 4.1. PaPaS Scale

The PaPaS Scale is a multidimensional tool developed to facilitate the timely and appropriate referral of children between 1 and 18 years old. The domains and items of this tool were established through a qualitative study adopting semi-structured interviews with international experts in PPC, followed by a focus group discussion [6]. In its current formulation, the PaPaS scale considers five domains for identifying children eligible for PPC, namely: (i) the trajectory of the disease and impact on daily activities; (ii) expected outcome and burden of treatment; (iii) symptom burden; (iv) preferences of patient, parents, and HCPs; and (v) life expectancy. Each domain includes two to five questions (items) for a total of 13 items. Each item presents at least two response options corresponding to different scores (0–4). The higher the total score, the greater the need for PPC; patients are then stratified into different courses of action: (1) no PC needed (score 10–14), (2) PC considered (15–24), and (3) PC needed (≥25) [6,7]. To further validate the PaPaS Scale, the investigators evaluated predictors of the need for PPC using a combination of statistical analysis and case vignette evaluation [7]. Life expectancy and child/family preferences were the strongest and most urgent indicators for PPC, allowing further scale refinement.

Each of the PaPaS Scale’s five domains includes several questions, comprising the ‘surprise question’, ‘Would you be surprised if this child were to die suddenly in 6 months?’, within the life expectancy domain. Each question is weighted and scored according to the answer. The total score is then used to stratify patients into different action programs; a score ≥ 15 means that PPC can be established [6,7]. This scale is available in English and German.

### 4.2. ACCAPED Scale

The ACCAPED Scale is a multidimensional questionnaire designed to assess the clinical needs of a child with a life-limiting or life-threatening illness. It was developed in Italian by a multidisciplinary group of PPC experts and was validated by the author by evaluating clinical vignettes [8,9]. An English version of the questionnaire is also available, although not yet validated.

In more detail, the ACCAPED Scale questionnaire investigates 10 domains of PPC needs: respiration, feeding, seizures/altered consciousness, skin integrity, mobility, communication, rest and sleep, continence and evacuation, drug administration, and pain. For each area, the most relevant needs are identified by a score that increases with the complexity of the assistance required. A modified version of the ‘surprise question’ (see below [10,11,12]) is also included and accounts for the maximum points when answered affirmatively. The final score is obtained from each domain, and the ‘surprise question’ scores guide the HCPs in determining the most appropriate PPC dimension for the patient. The child is referred to community care in the case of low-complexity needs (score ≤ 29), to the general PPC service in the case of medium-complexity needs (score 30–49), or to a specialized PPC service when high-complexity needs are involved (score ≥ 50). Furthermore, the ‘surprise question’ is considered effective for early referral to a specialist PC as a prognostic tool. Several formulations of the ‘surprise question’ have been provided, and the most frequently used is ’Would you be surprised if this child died in the next 12 months?’ [10,11,12].

## 5. Patient and Family Needs Assessment Tools

Patients in PPC and their families have multiple and interrelated physical, psychological, social, spiritual, and ethical needs whose intensity and importance change over time [2]. These needs should be continuously assessed throughout the disease trajectory using different tools, which can be general, i.e., assessing several needs by a single instrument, or specific, i.e., assessing one or a few specific need(s), such as pain (Table 3 and Table 4). We provide below the description of retrieved tools for assessing patient and family needs, according to their classification as general or specific tools.

### 5.1. General Tools

#### 5.1.1. The Parent and Child Needs Survey

The Parent and Child Needs Survey (PCNeeds) incorporates aspects such as the parent–child relationship, the illness’s impact on the family, and unique decision-making needs [13]. It was adapted from the Needs at the End-of-Life Screening Tool validated for adult PC populations [32] and was developed with the collaboration of PPC experts, multidisciplinary HCPs, and bereaved parents. It is structured in a concise but comprehensive form of 22 items, intentionally written for elementary reading, in which respondents are asked to rate their level of need on an 11-point scale (0–10) ranging from minimum to maximum estimates of need [13]. In addition, a comment space is provided after each item for parents to enter any additional information. After an initial formulation, it was refined by interviewing parents of children in PPC and was rated as an easy-to-complete instrument. A certified translation is available for Spanish. The developers reported promising psychometrics, although further reliability and validity study is needed on a wider and diverse set of respondents.

#### 5.1.2. Pediatric Palliative Care Early Intervention Tool

This is a nurse-driven evidence-based instrument developed and preliminarily tested [14]. It was proposed as a pediatric symptom inventory, divided into three main domain sections (diagnostic criteria, present and poorly controlled symptoms, distress), to document and quantify a patient’s symptom profile in the context of a life-threatening or life-limiting illness [14].

Through this tool, bedside nurses can identify patients with an uncontrolled symptom burden within several areas (pain, secretions, dyspnea, intractable seizures, nausea, vomiting, constipation, diarrhea, anorexia, cachexia, sleep disturbance, lethargy, anxiety, depression, and/or agitation), thereby potentially reducing the time between the onset of excessive symptom severity and the initiation of proper management.

#### 5.1.3. Family Empowerment Scale

Empowerment is defined as a process that enables a person to gain greater control over their life and make decisions to shape it [33]. This concept can also be applied to the status of parents of children coping with illness. Parental empowerment can be measured using the Family Empowerment Scale (FES), a 24-item self-reported tool available in English and Dutch [15]. This instrument was originally developed for the parents of children with emotional disabilities [34], but it has also been validated for parents of children with chronic conditions requiring PPC. The questionnaire provides insights into parents’ sense of empowerment. It consists of two domains, family and service systems: the former dimension reflects empowerment concerning the family, service system, and larger community and political environment; the latter describes the expression of empowerment in terms of attitudes, knowledge, and behaviors.

### 5.2. Specific Tools

Children in PPC present a plethora of complex physical and psychological symptoms, including pain, secretions, dyspnea, intractable convulsions, nausea, vomiting, constipation, diarrhea, anorexia, cachexia, sleep disturbances, asthenia, anxiety, depression, and agitation. In line with this principle, it would be desirable to have specific tools for each of these symptoms to measure their intensity when needed along the disease trajectory [2]. Indeed, several tools are used for this goal, but they have often been developed in settings other than PPC or are intended mainly for research purposes.

An excellent systematic review of tools used to measure the self-reported intensity of symptoms, specifically in oncology, was published by Pinheiro et al. [35]. The authors identified 38 instruments investigating physical and psychological symptoms, including pain, sleep quality, fatigue, nausea/vomiting, itching, anorexia, depression, and anxiety. A detailed description of each of these instruments can be found in the paper by Pinheiro et al. However, those that can be considered specific are listed in Table 4.

More recently, Chan et al. systematically identified pain assessment tools currently used in PPC [5]. Overall, they identified 22 pain assessment tools, among which the Faces Pain Scale-Revised (FPS-R); the Face, Legs, Activity, Cry, and Consolability (FLACC) scale; and the Pediatric Pain Profile (PPP) emerged as those endowed with higher internal consistency, criterion validity, reliability, and responsiveness (Table 4). The FPS-R is a self-assessment tool that consists of six drawings of faces and displays a neutral face on the left side (score 0) and a face showing maximum pain on the right side (score 10) [36]. The tool has been validated in patients aged 5–17 with oncological disease. Despite its simple use, cultural validation of this tool is still lacking. With the FLACC scale, originally developed for the assessment of postoperative pain, an external evaluator measures the child’s pain intensity by rating five behaviors (face, legs, activity, consolability, and cry) on a 0–2 scale to derive a total score from 0 to 10 [30]. Last, PPP is a 20-item scale in which an external evaluator rates each item on a 4-point scale [28]. This tool has been validated in children with neurological and cognitive impairment.

Remarkably, some symptoms frequently experienced by children in PPC are particularly challenging to manage. In particular, a recent project identified neuro-irritability, dystonia, and sleep disorders as the most difficult to manage [37]. Recommendations for their management are being developed through a Delphi approach, and the development of dedicated assessment tools will be a priority.

Religious and spiritual practices are immediately associated with the child and family’s cultural background and strongly influence how PPC is perceived [38]. In this specific field, McEvoy proposed a system called ‘B-E-L-I-E-F’, which can guide the assessment of the spiritual and religious needs of the child and family [39,40]. The letters in the acronym indicate the following: B—belief system, E—ethics or values, L—lifestyle, I—involvement in a spiritual community, E—education, and F—future events. These dimensions should be explored in assessing the religious and spiritual needs of the child and family.

## 6. Care Assessment Tools

Although objective measurement of the treatment effectiveness and quality of PPC services is considered a priority, only a few tools with this aim are currently available. Indeed, defining appropriate measures for PPC outcomes is challenging because of the heterogeneity of the child’s disease and conditions [3,41,42,43].

In our opinion, two tools are worth particular attention. Downing et al. developed a multidimensional outcome tool for PPC, the African C-POS, across eight African countries [3]. It comprises 12 questions to assess the QoL and PPC service (eight for the child or caregiver if the child cannot answer, and four for caregivers), each rated on a six-point Likert scale. Other versions of this tool are being developed in the UK and Belgium [44,45]. Another outcome measure, the FACETS-OF-PPC, specifically addressed to patients with severe neurological impairment, was developed by Pelke et al. in German and English [41]. This questionnaire, consisting of 53 questions rated on a six-point Likert scale, investigates caregivers’ current situation, the presence of symptoms, and the status and feelings of caregivers; it was developed using a rigorous approach and was validated by a multicenter study involving more than 300 caregivers/HCPs. A version for professional caregivers also exists.

Another tool, still under validation, is the Quality of Children’s Palliative Care Instrument (QCPCI), specifically targeting patients with oncological disease. It was preliminary developed by adapting a previous instrument and interviewing 533 parents [46].

Other tools published before 2019 were reviewed by Friedel et al. through a systematic search and quality assessment [42]. Specifically, they identified five tools most frequently used in the assessment of PPC quality: the Pediatric Quality of Life (PedsQL™) 4.0 [47], the Quality of Life in Life-Threatening Illness–Family Caregiver Questionnaire (QOLLTI–F) [48], the Supportive Care sCore (SCC) [49], the Needs at the End-of-Life Screening Tool (NEST) [50], and the Hospital Anxiety and Depression Scale (HADS) [48].

QoL is a proxy for the effectiveness of PPC services [2,43]. A systematic review published in 2016 discussed health-related QoL outcome measures with potential use in PPC (Table 5) [51]. It pointed out that the quality of the instruments varied widely and that many applied only to children with a specific disease. For instance, none of the tools listed in Table 5 were developed or validated in a PPC setting.

End-of-life care is a major component of PPC [2]. Hence, the assessment of end-of-life care is of utmost importance for the proper assessment of the quality of PPC. Zimmerman et al. developed and tested the Parental PELICAN Questionnaire, a tool that retrospectively assesses parental experiences and needs during their child’s end-of-life care [80]. This questionnaire includes approximately 90 items in four different versions specific to cardiology, neonatology, neurology, and oncology settings.

## 7. Concluding Remarks

In PPC, the adequate and continuous assessment of child and family needs and quality of care is fundamental. However, this assessment can be challenging due to the heterogeneity of patients in terms of age, developmental level, and clinical status. Furthermore, PPC is multidimensional and encompasses physical, psychological, social, and spiritual needs. Therefore, in current clinical practice, there is a major need to identify and apply validated tools with different aims: referring the patient to the most appropriate PPC service, assessing the severity of child and family needs, and monitoring the quality of PPC.

Our analysis of the currently available tools for PPC, although narrative, revealed that several instruments exist to assess the different dimensions of PPC. We also proposed a classification of these tools into eligibility tools, patient and family needs assessment tools, and PPC care assessment tools.

Referring patients to the proper service is the gateway for the PPC trajectory and is very important in ensuring the best quality of care and saving resources. At present, two main tools exist, the PaPaS Scale and the ACCAPED Scale questionnaire. Both tools seem to hold great relevance in daily practice, and they should always be applied at the first assessment of every patient. Remarkably, they were developed specifically in the PPC setting. The second group, i.e., the tools aimed at assessing symptom intensity, appears to be the most numerous. There are tools for the general assessment of symptoms or for investigating specific symptoms. However, it should be noted that most of the tools in this group have not been specifically validated in the PPC setting, and many may be more readily applied in research settings rather than in daily practice. Similar considerations can be made for tools assessing QoL, while tools assessing PPC service quality seem to be easily applied.

It is quite straightforward to understand that the ideal tool should be developed—or at least validated—in the PPC population, be validated in several languages, and be as general as possible for an immediate application in clinical practice. At present, not all available tools for PPC assessment meet these standards, and efforts to develop new specific tools and validate existing ones are undoubtedly advocated, as well as the need to deepen and expand the research in this area as a source for the definition of new clinical tools. The definition of tools to be used in clinical practice should take into account the importance of some characteristics, such as the ease of interpretation, use, and administration; the free accessibility; the exhaustiveness in terms of indications for age and pathology; and the non-invasiveness for patients and families. From the point of view of HCPs, the ideal tool should be as inexpensive as possible in terms of time and resources dedicated to the acquisition of information, should provide the possibility of remote administration and/or self-administration once the methods and objectives have been explained and shared, and should be repeatable over time and with easy-to-analyze outcomes, with the definition of net references that can clearly define the interpretation of the answers, even by HCPs non-specifically competent in the investigated field. These objectives are surely not easy to achieve, but the definition of the priority characteristics can be of support in the definition and in the choice of the tools to be used.

At the same time, we believe that objective assessment is imperative in PPC, in the patient’s best interest; to this end, HCPs working in this setting should start using available tools, regardless of their validation status. In particular, in the peculiar heterogeneity of the situations in PPC, each team should choose some tools (ideally one or more for each area) according to the clinical setting and available resources. The defined tools must be studied and known in-depth, shared, and applied systematically during clinical practice, with the constant updating of the medical record regarding the results and the constant team sharing and discussion about the objectives achieved. This work is useful to continuously evaluate the quality of the provided service and to identify critical issues and gaps to be solved. It is also necessary for the growth and implementation of services, as well as for the possibility to certify the work performed and to justify the request for support from institutions or other resources.

In the same way, it is important to deal with the training of pediatricians in this area, which is often neglected due to objectives and strategies adopted and/or choices of work priorities. However, it is increasingly mandatory for all professionals to acquire this attitude and skills.

Our review presents several limitations, such as the non-systematic criteria adopted for the literature search, the potential bias derived from the selection of paper based on relevance as judged by the authors, and the absence of a formal assessment of the tools’ quality, which was due to the wide heterogeneity of the tools themselves in terms of general structure and intent. However, to the best of our knowledge, this is the first attempt to provide a comprehensive overview of available tools for PPC, along with a classification to facilitate their application in clinical practice by HPC operating in this field.

## Figures and Tables

**Table 1:** Classification of assessment tools in pediatric palliative care.

Type of Tools	General Characteristics	Time of Application
Eligibility tools	They enable the early identification of children eligible for PPC by assessing their needs and helping refer them to the best possible PPC service	Initiation of PPC, but also along the disease trajectory
Patient and family needs assessment tools	They help assess the severity and management of the child’s and family’s needs These tools can be - General: they assess several needs using a single tool - Specific: they assess a specific need or symptom	Along the entire disease trajectory
Care assessment tools	They evaluate the quality of services and the effectiveness of the PPC programs	Along the entire disease trajectory

PPC: Pediatric palliative care.

**Table 2:** Main eligibility tools in pediatric palliative care.

Tool	Aim and Brief Description	Developed and/or Validated Specifically in PPC?
PaPaS Scale [6,7]	It assesses the burden of care for the child and caregivers and enables prompt referral to PPC service It consists of five domains. A score of ≥15 indicates that referral to PPC is needed	Yes
ACCAPED Scale [8,9]	It assesses the clinical needs of a child with a life-limiting or life-threatening illness It investigates 10 domains of PPC needs. The child is referred to community care in the case of low-complexity needs (score ≤ 29), to general PPC service in the case of medium-complexity needs (score 30–49), or to a specialized PPC service when high-complexity needs are present (score ≥ 50)	Yes
Surprise question [10,11,12]	If the reply to the question ‘Would you be surprised if this patient died in the next 12 months?’ is yes, the patient may be considered for PPC	No

PPC: Pediatric palliative care.

**Table 3:** Main patient and family needs’ assessment tools in pediatric palliative care: general tools.

Tool	Aim and Brief Description	Developed and/or Validated Specifically in PPC?
PCNeeds [13]	This patient- and parent-oriented scale assesses the severity of a range of needs using an 11-point scale and a comment space for each item	Yes
Pediatric Palliative Care Early Intervention Tool [14]	This is a nurse-driven instrument divided into three main domain sections that quantify the profile and severity of a range of symptoms	Yes
Family Empowerment Scale [15]	This 24-item self-reported tool provides insights into parents’ sense of empowerment	Yes

PPC: Pediatric palliative care.

**Table 4:** Main patient and family needs assessment tools in pediatric palliative care: specific tools.

Tool	Symptom Addressed	Developed and/or Validated Specifically in PPC?
Fatigue		
Fatigue Scale-Adolescents [16]	Fatigue	No
Fatigue Scale-Children [17]	Fatigue	No
**Gastrointestinal symptoms**		
10-cm Self-Report Visual Analog Scale Nausea [18]	Nausea	No
Constipation Assessment Scale [19]	Abdominal distension (bloating), constipation, diarrhea, fecal incontinence, rectal pain, change in flatulence	No
Oral Mucositis Daily Questionnaire [20,21]	Mouth and throat soreness, diarrhea, overall health	No
Pediatric Nausea Assessment Tool [22]	Nausea	No
**Mucositis**		
Children’s International Mucositis Evaluation Scale [23]	Mucositis (mouth/throat pain, dysphagia, mouth sores)	No
**Psychiatric symptoms**		
10-cm Self-Report Visual Analog Scale Anxiety [18]	Anxiety	No
Children’s Depression Inventory [24]	Depression	No
Revised Children’s Manifest Anxiety Scale [25]	Physiological anxiety (such as nausea, fatigue, insomnia, dyspnea), worry and oversensitivity (such as nervousness, fear, irritability, loneliness), concentration anxiety (such as restlessness)	No
State-Trait Anxiety Inventory for Children [26]	Apprehension, tension, worry	No
**Pain**		
Faces Pain Scale-Revised [27]	Pain	No
Pediatric Pain Profile [28]	Pain	No
Pain Squad App [29]	Pain	No
The Face, Legs, Activity, Cry and Consolability pain assessment tool [30]	Pain	No
**Sleep quality**		
Adolescent Sleep Wake Scale [31]	Sleep quality	No

PPC: Pediatric palliative care.

**Table 5:** Main tools for the quality-of-life assessment in PPC. Source: Adapted from Coombs et al. 2016 [51] (CC BY-NC 4.0 license).

Tool	Type of Report	Age of Application
Generic		
KIDSCREEN-52 [52]	Self-reported Parent	8–18 years
PedsQL™ 4.0 Generic Core Scales [53,54,55,56,57,58,59,60] Acute and standard version	Self-reported Parent	Toddlers (2–4 years), young children (5–7 years), children (8–12 years), adolescents (13–18 years)
**Cancer**		
Memorial Symptom Assessment Scale [61,62] 7–12 10–18	Self-reported	7–12 years 10–18 years
Pediatric Cancer Quality of Life Inventory (PCQL-32) [63,64]	Self-reported Parent	8–18 years
Pediatric Oncology Quality of Life Scale [65]	Parent	Adults
PedsQL™ Cancer Module [66] Acute and standard version	Self-reported Parent	Toddlers (2–4 years), young children (5–7 years), children (8–12 years), adolescents (13–18 years)
Royal Marsden Hospital Pediatric Oncology Quality of Life Questionnaire [67]	Parent	Adults
PedsQL™ Brain Tumor Module [68] Acute and standard version	Self-reported Parent	Toddlers (2–4 years), young children (5–7 years), children (8–12 years), adolescents (13–18 years)
**Cardiac disease**		
Pediatric Cardiac Quality of Life Inventory [69,70]	Self-reported Parent	Children (8–12 years) and adolescents (13–18 years)
PedsQL™ 3.0 Cardiac Module [71] Acute and standard version	Self-reported Parent	Toddlers (2–4 years), young children (5–7 years), children (8–12 years), adolescents (13–18 years)
**Neurological disease**		
Glasgow Epilepsy Outcome Scale for Young Persons (GEOSYP) [72]	Self-reported	Adolescents
Health-related quality of life in children with epilepsy [73]	Self-reported Parent	>8 years
PedsQL™ Cerebral Palsy Module [74] Acute and standard version	Self-reported Parent	Toddlers (2–4 years), young children (5–7 years), children (8–12 years), adolescents (13–18 years)
PedsQL™ Neuromuscular Module [75,76,77] Acute and standard version	Self-reported Parent	Toddlers (2–4 years), young children (5–7 years), children (8–12 years), adolescents (13–18 years)
Quality of Life in Childhood Epilepsy Questionnaire [78,79]	Parent	Adult

## Data Availability

Not applicable.

## References

[1] World Health Organization (1998). Cancer Pain Relief and Palliative Care in Children.

[2] Benini F., Pappadatou D., Bernadá M., Craig F., De Zen L., Downing J., Drake R., Friedrichsdorf S., Garros D., Giacomelli L. (2022). International Standards for Pediatric Palliative Care: From IMPaCCT to GO-PPaCS. J. Pain Symptom Manag..

[3] Downing J., Namisango E., Harding R. (2018). Outcome measurement in paediatric palliative care: Lessons from the past and future developments. Ann. Palliat. Med..

[4] National Consensus Project for Quality Palliative Care (2018). Clinical Practice Guidelines for Quality Palliative Care.

[5] Chan A.Y., Ge M., Harrop E., Johnson M., Oulton K., Skene S.S., Wong I.C., Jamieson L., Howard R.F., Liossi C. (2021). Pain assessment tools in paediatric palliative care: A systematic review of psychometric properties and recommendations for clinical practice. Palliat. Med..

[6] Bergstraesser E., Hain R.D., Pereira J.L. (2013). The development of an instrument that can identify children with palliative care needs: The Paediatric Palliative Screening Scale (PaPaS Scale): A qualitative study approach. BMC Palliat. Care.

[7] Bergstraesser E., Paul M., Rufibach K., Hain R.D., Held L. (2014). The Paediatric Palliative Screening Scale: Further validity testing. Palliat. Med..

[8] Lazzarin P., Giacomelli L., Biscaglia L., Terrenato I., Benini F. (2019). La valutazione della complessità assistenziale in cure palliative pediatriche La scala ACCAPED come strumento per la valutazione dei bisogni. Riv. Ital. Cure Palliat..

[9] Lazzarin P., Giacomelli L., Terrenato I., Benini F., Agosto C., Divisic A., Francesca R., Nicoletta M., Monica P., Catalano I. (2021). A Tool for the Evaluation of Clinical Needs and Eligibility to Pediatric Palliative Care: The Validation of the ACCAPED Scale. J. Palliat. Med..

[10] Jennings K.S., Marks S., Lum H.D. (2018). The Surprise Question as a Prognostic Tool #360. J. Palliat. Med..

[11] Downar J., Goldman R., Pinto R., Englesakis M., Adhikari N.K. (2017). The “surprise question” for predicting death in seriously ill patients: A systematic review and meta-analysis. Can. Med. Assoc. J..

[12] Burke K., Coombes L.H., Menezes A., Anderson A.-K. (2017). The ‘surprise’ question in paediatric palliative care: A prospective cohort study. Palliat. Med..

[13] Donnelly J.P., Downing K., Cloen J., Fragen P., Gupton A.W., Misasi J., Michelson K. (2018). Development and Assessment of a Measure of Parent and Child Needs in Pediatric Palliative Care. J. Pain Symptom Manag..

[14] Shaw R.M., Seegal H.M., Miller J.G.M., Keim-Malpass J. (2018). Pilot of a Pediatric Palliative Care Early Intervention Instrument. J. Hosp. Palliat. Nurs..

[15] Segers E.W., van den Hoogen A., van Eerden I.C. (2018). Perspectives of parents and nurses on the content validity of the Family Empowerment Scale for parents of children with a chronic condition: A mixed-methods study. Child Care Health Dev..

[16] Hinds P.S., Hockenberry M., Tong X., Rai S.N., Gattuso J.S., McCarthy K., Pui C.H., Srivastava D.K. (2007). Validity and reliability of a new instrument to measure cancer-related fatigue in adolescents. J. Pain Symptom Manag..

[17] Hinds P.S., Yang J., Gattuso J.S., Hockenberry M., Jones H., Zupanec S., Li C., Crabtree V.M., Mandrell B.N., Schoumacher R.A. (2010). Psychometric and clinical assessment of the 10-item reduced version of the Fatigue Scale-Child instrument. J. Pain Symptom Manag..

[18] Redd W.H., Jacobsen P.B., Die-Trill M., Dermatis H., McEvoy M., Holland J.C. (1987). Cognitive/attentional distraction in the control of conditioned nausea in pediatric cancer patients receiving chemotherapy. J. Consult. Clin. Psychol..

[19] Woolery M., Carroll E., Fenn E., Wieland H., Jarosinski P., Corey B., Wallen G.R. (2006). A constipation assessment scale for use in pediatric oncology. J. Pediatr. Oncol. Nurs..

[20] Manji A., Tomlinson D., Ethier M.C., Gassas A., Maloney A.M., Sung L. (2012). Psychometric properties of the Oral Mucositis Daily Questionnaire for child self-report and importance of mucositis in children treated with chemotherapy. Support. Care Cancer..

[21] Tomlinson D., Isitt J.J., Barron R.L., Doyle J., Judd P., Gassas A., Naqvi A., Sung L. (2008). Determining the understandability and acceptability of an oral mucositis daily questionnaire. J. Pediatr. Oncol. Nurs..

[22] Dupuis L.L., Taddio A., Kerr E.N., Kelly A., MacKeigan L. (2006). Development and validation of the pediatric nausea assessment tool for use in children receiving antineoplastic agents. Pharmacotherapy.

[23] Jacobs S., Baggott C., Agarwal R., Hesser T., Schechter T., Judd P., Tomlinson D., Beyene J., Sung L. (2013). Validation of the Children’s International Mucositis Evaluation Scale (ChIMES) in paediatric cancer and SCT. Br. J. Cancer.

[24] Mulhern R.K., Fairclough D., Douglas S.M., Smith B. (1994). Physical Distress and Depressive Symptomatology among Children with Cancer. Child. Health Care.

[25] Reynolds C.R., Richmond B.O. (1985). The Revised Children’s Manifest Anxiety Scale: Manual.

[26] Jacobs S., Mowbray C., Cates L.M., Baylor A., Gable C., Skora E., Estrada M., Cheng Y., Wang J., Lewin D. (2016). Pilot Study of Massage to Improve Sleep and Fatigue in Hospitalized Adolescents with Cancer. Pediatr. Blood Cancer.

[27] Fortier M.A., Wahi A., Bruce C., Maurer E.L., Stevenson R. (2013). Pain management at home in children with cancer: A daily diary study. Pediatr. Blood Cancer.

[28] Hunt A., Goldman A., Seers K., Crichton N., Mastroyannopoulou K., Moffat V., Oulton K., Brady M. (2004). Clinical validation of the paediatric pain profile. Dev. Med. Child Neurol..

[29] Stinson J.N., Jibb L.A., Nguyen C., Nathan P.C., Maloney A.M., Dupuis L.L., Gerstle J.T., Hopyan S., Alman B.A., Strahlendorf C. (2015). Construct validity and reliability of a real-time multidimensional smartphone app to assess pain in children and adolescents with cancer. Pain.

[30] Merkel S., Voepel-Lewis T., Shayevitz J.R., Malviya S. (1997). The FLACC: A behavioral scale for scoring postoperative pain in young children. Pediatr. Nurs..

[31] Walker A.J., Johnson K.P., Miaskowski C., Lee K.A., Gedaly-Duff V. (2010). Sleep Quality and Sleep Hygiene Behaviors of Adolescents during Chemotherapy. J. Clin. Sleep Med..

[32] Emanuel L.L., Alpert H.R., Emanuel E.E. (2001). Concise Screening Questions for Clinical Assessments of Terminal Care: The Needs Near the End-of-Life Care Screening Tool. J. Palliat. Med..

[33] Herbert R.J., Gagnon A.J., Rennick J.E., O’loughlin J.L. (2009). A Systematic Review of Questionnaires Measuring Health-Related Empowerment. Res. Theory Nurs. Pract..

[34] Koren P.E., DeChillo N., Friesen B.J. (1992). Measuring empowerment in families whose children have emotional disabilities: A brief questionnaire. Rehab. Psychol..

[35] Pinheiro L.C., McFatrich M., Lucas N., Walker J.S., Withycombe J.S., Hinds P.S., Sung L., Tomlinson D., Freyer D.R., Mack J.W. (2017). Child and adolescent self-report symptom measurement in pediatric oncology research: A systematic literature review. Qual. Life Res..

[36] Hicks C.L., von Baeyer C.L., Spafford P.A., van Korlaar I., Goodenough B. (2001). The Faces Pain Scale—Revised: Toward a common metric in pediatric pain measurement. Pain.

[37] Avagnina I., Giacomelli L., Mercante A., Benini F. (2023). International project on troublesome symptoms in paediatric palliative care will focus on neuro-irritability, dystonia and sleep disorders. Acta Paediatr..

[38] Givler A., Bhatt H., Maani-Fogelman P.A. (2021). The importance of cultural competence in pain and palliative care. StatPearls.

[39] Amery J., Garanganga E., Horne C., Amery J. (2009). Spirituality in Children’s Palliative Care in Africa.

[40] McEvoy M. (2006). An added dimension to the pediatric health maintenance visit: The spiritual history. J. Pediatr. Health Care.

[41] Pelke S., Wager J., Claus B.B., Zernikow B., Reuther M. (2021). Development and psychometric validation of the family-centered multidimensional outcome measure for pediatric palliative care targeted to children with severe neurological impairmentis—A multicenter prospective study. Palliat. Med..

[42] Friedel M., Aujoulat I., Dubois A.-C., Degryse J.-M. (2019). Instruments to Measure Outcomes in Pediatric Palliative Care: A Systematic Review. Pediatrics.

[43] Boyden J.Y., Bogetz J.F., Johnston E.E., Thienprayoon R., Williams C.S., McNeil M.J., Patneaude A., Widger K.A., Rosenberg A.R., Ananth P. (2023). Measuring Pediatric Palliative Care Quality: Challenges and Opportunities. J. Pain Symptom Manag..

[44] Namisango E., Downing J. Progress in the development of a Children’s Palliative Care Outcome Scale. Proceedings of the Open Meeting EAPC Task Force Children and Young People, 11th World Research Congress.

[45] Friedel M., Brichard B., Boonen S., Tonon C., De Terwangne B., Bellis D., Mevisse M., Fonteyne C., Jaspard M., Schruse M. (2021). Face and content validity, acceptability and feasibility of the adapted version of the Children’s Pallaitive Outcome Scale: A qualitative Pilot Study. J. Palliat. Med..

[46] Widger K., Brennenstuhl S., Duc J., Tourangeau A., Rapoport A. (2019). Factor structure of the Quality of Children’s Palliative Care Instrument (QCPCI) when completed by parents of children with cancer. BMC Palliat. Care.

[47] Wolfe J., Orellana L., Cook E.F., Ullrich C., Kang T., Geyer J.R., Feudtner C., Weeks J.C., Dussel V. (2014). Improving the Care of Children with Advanced Cancer by Using an Electronic Patient-Reported Feedback Intervention: Results from the PediQUEST Randomized Controlled Trial. J. Clin. Oncol..

[48] Groh G., Borasio G.D., Nickolay C., Bender H.U., von Lüttichau I., Führer M. (2013). Specialized Pediatric Palliative Home Care: A Prospective Evaluation. J. Palliat. Med..

[49] Friedrichsdorf S.J., Postier A., Dreyfus J., Osenga K., Sencer S., Wolfe J. (2015). Improved Quality of Life at End of Life Related to Home-Based Palliative Care in Children with Cancer. J. Palliat. Med..

[50] Al-Gharib R.M., Huijer H.A.-S., Darwish H. (2015). Quality of care and relationships as reported by children with cancer and their parents. Ann. Palliat. Med..

[51] Coombes L.H., Wiseman T., Lucas G., Sangha A., Murtagh F.E. (2016). Health-related quality-of-life outcome measures in paediatric palliative care: A systematic review of psychometric properties and feasibility of use. Palliat. Med..

[52] Erhart M., Ravens-Sieberer U., Dickinson H.O., Colver A., European SPARCLE and KIDSCREEN Groups (2009). Rasch Measurement Properties of the KIDSCREEN Quality of Life Instrument in Children with Cerebral Palsy and Differential Item Functioning between Children with and without Cerebral Palsy. Value Health.

[53] Varni J.W., Seid M., Rode C.A. (1999). The PedsQL™: Measurement Model for the Pediatric Quality of Life Inventory. Med. Care.

[54] Varni J.W., Burwinkle T.M., Seid M., Skarr D. (2003). The PedsQL 4.0 as a pediatric population health measure: Feasibility, reliability, and validity. Ambul. Pediatr..

[55] Varni J.W., Limbers C.A., Burwinkle T.M. (2007). How young can children reliably and validly self-report their health-related quality of life?: An analysis of 8,591 children across age subgroups with the PedsQL™ 4.0 Generic Core Scales. Health Qual. Life Outcomes.

[56] Varni J., Limbers C., Burwinkle T. (2007). Parent proxy-report of their children’s health-related quality of life: An analysis of 13,878 parents’ reliability and validity across age subgroups using the PedsQL™ 4.0 Generic Core Scales. Health Qual. Life Outcomes.

[57] Varni J.W., Limbers C.A., Newman D.A. (2008). Factorial Invariance of the PedsQL™ 4.0 Generic Core Scales Child Self-Report Across Gender: A Multigroup Confirmatory Factor Analysis with 11,356 Children Ages 5 to 18. Appl. Res. Qual. Life.

[58] Varni J., Limbers C., Newman D. (2009). Using factor analysis to confirm the validity of children’s self-reported health-related quality of life across different modes of administration. Clin. Trials.

[59] Amin L., Rosenbaum P., Barr R., Sung L., Klaassen R.J., Dix D.B., Klassen A. (2012). Rasch analysis of the PedsQL: An increased understanding of the properties of a rating scale. J. Clin. Epidemiol..

[60] Huang I.-C., Shenkman E., Madden V.L., Vadaparampil S., Quinn G., Knapp C. (2010). Measuring quality of life in pediatric palliative care: Challenges and potential solutions. Palliat. Med..

[61] Collins J.J., Devine T.D., Dick G.S., Johnson E., Kilham H., Pinkerton C., Stevens M., Thaler H.T., Portenoy R.K. (2002). The Measurement of Symptoms in Young Children with Cancer: The Validation of the Memorial Symptom Assessment Scale in Children Aged 7–12. J. Pain Symptom Manag..

[62] Collins J.J., Byrnes M.E., Dunkel I.J., Lapin J., Nadel T., Thaler H.T., Polyak T., Rapkin B., Portenoy R.K. (2000). The Measurement of Symptoms in Children with Cancer. J. Pain Symptom Manag..

[63] Varni J.W., Katz E.R., Seid M., Friedman-Bender A., Quiggins D.J.L. (1998). The pediatric cancer quality of life inventory-32 (PCQL-32): I. Reliability and validity. Cancer.

[64] Seid M., Varni J.W., Rode C.A., Katz E.R. (1999). The Pediatric Cancer Quality of Life Inventory: A modular approach to measuring health-related quality of life in children with cancer. Int. J. Cancer.

[65] Goodwin D., Boggs S., Graham-Pole J. (1994). Development and validation of the pediatric oncology quality of life scale. Psychol. Assess..

[66] Varni J.W., Burwinkle T.M., Katz E.R., Meeske K., Dickinson P. (2002). The PedsQL in pediatric cancer. Cancer.

[67] Watson M., Edwards L., Von Essen L., Davidson J., Day R., Pinkerton R. (1999). Development of the Royal Marsden Hospital paediatric oncology quality of life questionnaire. Int. J. Cancer.

[68] Palmer S.N., Meeske K.A., Katz E.R., Burwinkle T.M., Varni J.W. (2007). The PedsQL brain tumor module: Initial reliability and validity. Pediatr. Blood Cancer.

[69] Marino B.S., Shera D., Wernovsky G., Tomlinson R.S., Aguirre A., Gallagher M., Lee A., Cho C.J., Stern W., Davis L. (2008). The development of the pediatric cardiac quality of life inventory: A quality of life measure for children and adolescents with heart disease. Qual. Life Res..

[70] Marino B.S., Tomlinson R.S., Wernovsky G., Drotar D., Newburger J.W., Mahony L., Mussatto K., Tong E., Cohen M., Andersen C. (2010). Validation of the Pediatric Cardiac Quality of Life Inventory. Pediatrics.

[71] Wray J., Franklin R., Brown K., Cassedy A., Marino B.S. (2013). Testing the Pediatric Cardiac Quality of Life Inventory in the United Kingdom. Acta Paediatr..

[72] Townshend K.H., Dorris L., McEwan M.J., Aylett S.E., Brodie M.J., O’regan M., Espie C. (2008). Development and validation of a measure of the impact of epilepsy on a young person’s quality of life: Glasgow epilepsy outcome scale for young persons (GEOS-YP). Epilepsy Behav..

[73] Ronen G.M., Streiner D.L., Rosenbaum P., Network C.P.E. (2003). Health-related Quality of Life in Children with Epilepsy: Development and Validation of Self-report and Parent Proxy Measures. Epilepsia.

[74] Varni J.W., Burwinkle T.M., Berrin S.J., Sherman S.A., Artavia K., Malcarne V.L., Chambers H.G. (2006). The PedsQL in pediatric cerebral palsy: Reliability, validity, and sensitivity of the Generic Core Scales and Cerebral Palsy Module. Dev. Med. Child Neurol..

[75] Iannaccone S.T., Hynan L.S., Morton A., Buchanan R., Limbers C.A., Varni J.W. (2009). The PedsQL in pediatric patients with Spinal Muscular Atrophy: Feasibility, reliability, and validity of the Pediatric Quality of Life Inventory Generic Core Scales and Neuromuscular Module. Neuromuscul. Disord..

[76] Davis S.E., Hynan L.S., Limbers C.A., Andersen C.M., Greene M.C., Varni J.W., Iannaccone S.T. (2010). The PedsQL in pediatric patients with Duchenne muscular dystrophy: Feasibility, reliability, and validity of the Pediatric Quality of Life Inventory Neuromuscular Module and Generic Core Scales. J. Clin. Neuromuscul. Dis..

[77] Dunaway S., Montes J., Montgomery M., Battista V., Koo B., Marra J., De Vivo D.C., Hynan L.S., Iannaccone S.T., Kaufmann P. (2010). Reliability of telephone administration of the PedsQL Generic Quality of Life Inventory and Neuromuscular Module in spinal muscular atrophy (SMA). Neuromuscul. Disord..

[78] Sabaz M., Cairns D.R., Lawson J.A., Nheu N., Bleasel A.F., Bye A.M. (2000). Validation of a New Quality of Life Measure for Children with Epilepsy. Epilepsia.

[79] Sabaz M., Lawson J.A., Cairns D.R., Duchowny M.S., Resnick T.J., Dean P.M., Bye A.M. (2003). Validation of the quality of life in childhood epilepsy questionnaire in American epilepsy patients. Epilepsy Behav..

[80] Zimmermann K., Cignacco E., Eskola K., Engberg S., Ramelet A.S., Von der Weid N., Bergstraesser E. (2015). Development and initial validation of the Parental PELICAN Questionnaire (PaPEQu): An instrument to assess parental experiences and needs during their child’s end-of-life care. J. Adv. Nurs..

